# Metabolic Modeling of *Pectobacterium parmentieri* SCC3193 Provides Insights into Metabolic Pathways of Plant Pathogenic Bacteria

**DOI:** 10.3390/microorganisms7040101

**Published:** 2019-04-05

**Authors:** Sabina Zoledowska, Luana Presta, Marco Fondi, Francesca Decorosi, Luciana Giovannetti, Alessio Mengoni, Ewa Lojkowska

**Affiliations:** 1 Department of Biotechnology, Intercollegiate Faculty of Biotechnology, University of Gdansk and Medical University of Gdansk, 58 Abrahama Street, 80-307 Gdansk, Poland; sabina.zoledowska@biotech.ug.edu.pl (S.Z.); ewa.lojkowska@biotech.ug.edu.pl (E.L.); 2 Department of Biology, University of Florence, via Madonna del Piano 6, Sesto Fiorentino, 50019 Florence, Italy; luana.presta@unifi.it (L.P.); marco.fondi@unifi.it (M.F.); 3 Department of Agri-food Production and Environmental Sciences, University of Florence, Piazzale delle Cascine 18, 50144 Florence, Italy; francesca.decorosi@unifi.it (F.D.); luciana.giovannetti@unifi.it (L.G.)

**Keywords:** Flux Balance Analysis, plant pathogenic bacteria, bacterial adaptation, metabolic reactions

## Abstract

Understanding plant–microbe interactions is crucial for improving plants’ productivity and protection. Constraint-based metabolic modeling is one of the possible ways to investigate the bacterial adaptation to different ecological niches and may give insights into the metabolic versatility of plant pathogenic bacteria. We reconstructed a raw metabolic model of the emerging plant pathogenic bacterium *Pectobacterium parmentieri* SCC3193 with the use of KBase. The model was curated by using inParanoind and phenotypic data generated with the use of the OmniLog system. Metabolic modeling was performed through COBRApy Toolbox v. 0.10.1. The curated metabolic model of *P. parmentieri* SCC3193 is highly reliable, as in silico obtained results overlapped up to 91% with experimental data on carbon utilization phenotypes. By mean of flux balance analysis (FBA), we predicted the metabolic adaptation of *P. parmentieri* SCC3193 to two different ecological niches, relevant for the persistence and plant colonization by this bacterium: soil and the rhizosphere. We performed in silico gene deletions to predict the set of essential core genes for this bacterium to grow in such environments. We anticipate that our metabolic model will be a valuable element for defining a set of metabolic targets to control infection and spreading of this plant pathogen.

## 1. Introduction

Plant–bacteria interplays have been studied for a long time, mainly regarding pathogenic and beneficial (symbiotic) interactions. Various details are now known concerning the molecular basis of all these interactions [1]. For example, biological studies of plant pathogenic bacteria allowed understanding the modulation of bacterial recognition by the plants and revealed important aspects of plant immune responses [2]. Furthermore, additional investigations have confirmed that plant pathogenic bacteria exploit high flexibility in utilization of different kinds of sugar, nitrogen, and phosphorus resources while adapting to the new environment; e.g., bacterial plant pathogen *Pseudomonas syringae* pv. *tomato* explicitly employs amino acid and sugar transporters to gain access to nutrients present in the environment [3]. Subsequently, during infection processes of the tomato plants, it utilizes resources within the host directly from the apoplast fluid [4].

To access the nutrients present in the plant tissues, pathogenic bacteria colonize, invade, and, later on, establish chronic infections within host plants. During the infection process, they enter plant tissues, through either wounds or natural openings, and occupy the apoplast of tissues or the xylem, where they multiply and spread [5]. Phytopathogenic microorganisms cause damage and often impair plant growth and reproduction. On the other hand, to defend themselves against microbiological invasion, plants rely on two kinds of innate immunity, i.e., via pathogen triggered immunity (PTI), which corresponds to pathogen perception via the recognition of conserved pathogen-associated molecular patterns and effector-triggered immunity (ETI) based on the recognition of pathogen effectors, molecules synthesized by the pathogens and delivered in the extracellular matrix or into the plant cell to enhance pathogen fitness [6].

To achieve compatible interaction, microorganisms at first need to overcome the plant’s defenses that could abort the infection. Plant pathogenic bacteria, like *Pectobacterium parmentieri*, combine numerous strategies to accomplish that goal; e.g., they rely on the quorum sensing system [7].

*P. parmentieri* are pectinolytic bacteria belonging to *Pectobacteriaceae* family (known as Soft Rot *Pectobacteriaceae*, SRP) [8,9], which are causative agents of soft rot in economically important crops such as potato, tomato, and maize [10]. Also, they are responsible for the blackleg disease, so far reported only on potato plants [11]. These Gram(-), rod-shaped bacteria are necrotrophs, able to destroy plant tissue components through the activity of Plant Cell Wall Degrading Enzymes (PCWDE) such as pectinases, cellulases, and proteases, secreted via Type I or II secretion system [12,13]. Interestingly, bacteria belonging to this species can produce different levels of the above-mentioned enzymes [14]. However, to cause disease symptoms those bacteria require favorable environmental conditions, such as humidity, nutrient availability accessed via natural plant openings, and preferably lower host resistance [11]. On the other hand, they can reside inside plant tissues as endophytes for a long time [11]. It is worth mentioning that bacteria from the genus *Pectobacterium* have been included among the ten most significant bacterial plant pathogens based on their economic impact [15] since crop losses caused by phytopathogenic microorganisms can reach up to 20% of total yield [16].

There is still limited knowledge regarding the cascade of genes expressed before and during the infection process in *P. parmentieri*. It was previously reported that initialization of infection progress is controlled by quorum sensing in closely related *Pectobacterium atrosepticum* [17]. Moreover, massive production of butanediol during plant infection by bacteria of the genera *Dickeya* and *Pectobacterium* was reported [18]. However, metabolic pathways necessary for promoting bacterial multiplication before and during plant infection connected with carbon and other compounds utilization in *P. parmentieri* have received little attention so far.

Given the complexity of bacteria–host relationships, they cannot be adequately investigated only using classical microbiological and molecular methods; instead, the coupled use of Phenotype Microarrays, computational, large-scale, and systematic frameworks are advisable. Constraint-based metabolic modeling is now a promising way to interpret puzzling, heterogeneous bacterial phenotypes [19], especially those related to bacteria–host interaction [20]. Constraint-based approaches, and in particular Flux Balance Analysis (FBA) [21], have been shown to infer realistic growth phenotypes and are claimed to provide a systems biology view on multi-omics data, possibly allowing prediction of physiological changes and evolution of bacterial populations [22,23]. Recently, genome-scale metabolic model (GEM) reconstruction and FBA have been used for deciphering the metabolic adaption of environmental microbes following ecological parameters variation [24], or comparison of activated metabolic reactions during ecological niche change [25], as well as for providing insights into the metabolic adaptation in human and bacterial plant pathogens [3,26]. To the best of our knowledge, only in a few cases, FBA has been applied in understanding the metabolic adaptation of specific plant bacterial pathogens. Studies performed on *Ralstonia solanacearum* showed that the quorum-sensing-dependent regulatory protein PhcA32 controls the trade-off between virulence factor production and bacterial proliferation [27]. Also, metabolic modeling of *P. carotovorum* subsp. *carotovorum* PC1 revealed 19 potential bactericide targets through a comprehensive in silico gene-deletion study [26].

The aim of this work was the reconstruction of a highly curated metabolic model of the plant pathogenic bacterium *P. parmentieri* SCC3193 and the exploitation of this model to putatively identify the metabolic pathways relevant for *P. parmentieri* fitness in two different ecological niches, i.e., soil and rhizosphere. We show that niche changes may lead to a metabolic reconstitution of carbon-related pathways in *P. parmentieri* SCC3193 and we spot the core-set of essential genes in the two examined conditions. Moreover, we anticipate the model itself will represent a valuable element, which will pave the way to both the knowledge-base of the strain’s biology and novel, applied technologies, such as genetic engineering and synthetic biology experiments.

## 2. Materials and Methods

### 2.1. Metabolic Network Reconstruction and Model Refinement

A draft metabolic model was built using the KBase Narrative Interface (www.kbase.com) which supports the reconstruction of metabolic models in microbes based on functional protein annotations. The closed genome of *Pectobacterium* sp. SCC3193 (NCBI Reference Sequence: NC_017845.1) [28] was used as an entry for such a process. The KBase automatic gap-filling algorithm was then run to improve the model. Automatic gap-filling compares the set of reactions in a given metabolic model to a database of all known reactions and finds a minimal set of reactions enabling it to grow in a predefined media, meaning that it forces a minimum flux of 0.1 through the biomass reaction. In our case, the medium growth chosen was Carbon–Glycerol, since its composition, (Supplementary Table S4 in Supplementary Information 2) is very similar to M9, one of the designed growth media of our experimental conditions.

This process allowed adding 44 new reactions and made two already existing reactions reversible (all the 46 reactions are listed in Supplementary Data 1). Afterwards, the model was downloaded and locally expanded by embedding reactions of orthologous genes present in the model of the closely related organism *Escherichia coli* K-12 MG1655 [29]; precisely, 93 genes and 383 reactions. The reason for this further model expansion is that the KBase gap-filling process may have discarded such reactions since they may not have been required for growth in Carbon–Glycerol. However, the strain may require fulfillment of these functions under different growth conditions.

Since the majority of *E. coli* reactions found belonged to a specific cellular district, the periplasm, we added this compartment in *P. parmentieri* model. The correctness of all the transporters added was verified during the last step of the model refinement, (i.e., the comparison of in silico outcomes to physiological abilities of the strain obtained through Phenotype Microarray (Omnilog) Experiment), where we observed that all the added gene allowed the model to grow on a different carbon source correctly.

Finally, during this test phase, the metabolic reconstruction was further enriched with exchange reactions (these reactions are the boundaries of the systems, representing the supply/removal of metabolites from the extra-organism space, so they do not need any biochemical or experimental evidence to be appended). At this stage, the model still presented six discrepancies that we decided to fix with manual gap-filling. Precisely, since PM showed evidence of strain growth on amylotriose, dihydroxyacetone, glucose-6-phosphate, d-arabinose, *N*-acetyl-d-galactosamine, stachyose, and sucrose, we added gap-filling transporters for such compounds, and we named them after the missing metabolite, even if the gene–protein relationship for that function was absent. These gap-fillings are fictitious reactions enabling the model to replicate a known physiological property of the strain when gene annotations for such a function are missing or when the metabolites enter the cell through one or more nonspecific transporter. The qualitative results of the comparison are reported in Supplementary Data 1 where, for each compound used as a carbon source, we show the growth predictions yielded by the model before and after the last round of refinements.

### 2.2. Biomass

No comprehensive description of the macromolecular composition of the *P. parmentieri* biomass is available in the literature. However, such data are available for *E. coli* [29] which, like *Parmentieri* genus, belongs to the *Enterobacteriaceae* family. Therefore, the generic Gram-negative biomass reaction embedded in the model by KBase was substituted with the more accurate and similar one of *E. coli* model [29] and set as the model’s objective function in all the experiments performed in this work. Despite being an approximation, that change gives a more realistic framework to perform simulations and it is allowed by strains of phylogenetic proximity and similar compound usage (as from Biolog outcomes comparison).

The complete biomass composition is given in Supplementary Information 2.

### 2.3. Metabolic Modeling

Metabolic modeling was performed using COBRApy Toolbox version 0.10.1 [30] and the Gurobi 7.0.2 solver (www.gurobi.com) with libSBML library v5.10, in Python 2.7 framework (Department of Bioengineering, University of California, San Diego, CA, USA). The scripts for running all the in silico analysis performed in this work are available in Supplementary Information 1.

### 2.4. Bacterial Strains and Culture Conditions

The bacterial strain used in this study is *P. parmentieri* reference strain SCC3193, isolated from potato tuber in Finland [9,31]. For high-throughput phenotypic characterization, bacteria were grown on TSA medium at 28 °C for 24 h. For EnVision™ experiment bacteria were first grown in LB at 28 °C for 24 h with constant agitation (120 RPM), later on in M9 for 24 h with constant agitation (130 RPM).

### 2.5. Experimental High-Throughput Phenotypic Characterization on P. parmentieri SCC3193

For high-throughput phenotypic characterization of *P. parmentieri* SCC3193 Biolog Plates for Carbon, Nitrogen, and Phosphorus-Sulfur utilization assay were used (PM1and PM2A, PM3, and PM4, respectively). Overnight bacterial culture was transferred from TSA medium to 5 mL of 0.85% NaCl, and bacterial suspension was adjusted to OD_600_ equaling 0.1 Later, 1 mL of bacterial suspension was transferred to 11 mL of Minimal Salts medium (M9-C: 0.6% Na_2_HPO_4_, 0.3% KH_2_PO_4_, 0.05% NaCl, 0.1 % NH_4_Cl, 0.005% Yeast Extract) supplemented with 120 µL of Biolog A dye. To inoculate wells in PM plates 100 µL of described bacterial suspension was used. The measurement was carried out in OmniLog™ for 46 h. Each reaction was tested in triplicate, and the experiment was performed twice. Mean results were analyzed with DuctApe [32]. All the results are reported in Supplementary Data 5.

Nutrients metabolic assay with the use of EnVision^TM^ plate reader was performed to cross-check high-throughput phenotypic characterization. M9 media supplemented with 20% of selected carbon sources: α-d-glucose, d-xylose, and d-mannitol, d-fructose, and citric acid were prepared. *P. parmentieri* SCC3193 was grown in LB medium overnight at 28 °C with constant agitation (120 RPM). Afterward, overnight bacterial cultures were centrifuged and washed twice in sterile Ringer Buffer and later OD_600_ of inoculum was adjusted to 0.1. Fifty µL of inoculum was transferred to 450 µL of M9 supplemented with different carbon sources to establish growth curves. Bacteria were cultured with agitation set at 120 RPM in 28 °C in 24-well plate in EnVision^TM^ plate reader. Optical density measurement at 600 nm was performed every 30 min. The quantification of wet bacterial biomass was performed after bacterial growth in 300 mL of the media mentioned above after 48 h. Bacterial cultures were centrifuged at 10 °C with 10,000 RPM for 10 min (Eppendorf Centrifuge 5920 R, Eppendorf Ltd., Warsaw, Poland) and frozen for 24 h. Afterward, the wet bacterial biomass was weighed.

### 2.6. OmniLog™ Data Processing and Analysis

PM1 and PM2A obtained data analysis was performed with DuctApe [32]. Activity index (AV) values were calculated following subtraction of the value obtained for blank well from that of inoculated wells, whereas plots of the growth curves are of the unblanked data. Bacterial growth with each compound was considered positive if the AV value was ≥3. Growth phenotypes were defined as negative if the AV value was ≤2 and following a manual inspection of the unblanked curves. All the results are reported in Supplementary Data 5.

### 2.7. In Silico Environmental Representations

In silico representations of the nutritional composition of the soil, and rhizosphere were derived from a previously published paper [25]. The composition of each ere medium used is reported in Supplementary Information 2 (M9, soil, and rhizosphere). The composition of carbon–glycerol medium used through the KBase gap-filling application is reported.

### 2.8. Flux Balance Analysis and Flux Variability Analysis

Flux distribution predictions were assessed by performing FBA in M9, soil, and rhizosphere media. We performed loopless-FBA to avoid net fluxes around a closed cycle in a network at steady state, known as loops.

Moreover, since FBA only predicts one flux distribution among all possible solutions, we also performed loopless-FVA (Flux Variability Analysis). Since in some cases, the range of reaction flux was unbounded (going from −1000 to 1000) we decided to filter out those reactions whose FVA predicted range was larger than the predicted FBA flux the following criteria:

(1) If f_FBA_ < 0

f_FVA_, min ≥ 1.2* f_FBA_ ˄ f_FVA_, max ≤ 0.8* f_FBA_

(2) If f_FBA_ > 0

f_FVA_, min ≥ 0.8* f_FBA_ ˄ f_FVA_, max ≤ 1.2* f_FBA_

Loopless FBA and FVA predictions for each reaction can be retrieved in Supplementary Data 3 or by running Supplementary Information 1.

### 2.9. Model’s Predictive Value Estimation

M9 growth medium was simulated in silico by constraining the lower bound of import reactions for each of the compounds present in the medium (as reported in Supplementary Information 2). When reproducing the PM experiment in silico, the model’s performances were considered as true positives (TP) if growth was obtained both in silico and in vivo, true negatives (TN) in case of nongrowth both in silico and in vivo, false positives (FP) if growth was obtained in silico but not in vivo, and false negatives (FN) if vice versa.

The reliability of the obtained predictions was then estimated according to the following parameters:

(3) Sensitivity = TP/(TP + FN)

(4) Specificity = TN/(TN + FP)

(5) Precision (PPV) = TP/(TP + FP)

(6) Negative predicted value (NPV) = TN/(TN + FN)

(7) Accuracy = (TP+TN)/(TP + TN + FP + FN)

(8) *F*-score = 2(precision × sensitivity)/(precision + sensitivity)

### 2.10. Single Gene Deletion Analysis

Using genome-scale metabolic networks (GEMs), gene knockouts can be simulated to identify those genes whose removal is likely to impair the organism’s growth. Specifically, it is possible to simulate mutants by deleting each gene included in the metabolic reconstruction and testing the predicted effects on the microbe’s growth. Through this strategy, it is possible to estimate the contribution of each gene to the fitness of the strain by calculating the growth ratio (GRratio) between the growth rate of the mutant model (*μ*_KO_) and the one of the wild-type (*μ*_WT_) as:

(9) GR_ratio_ = *μ*_KO_/*μ*_WT_

The knocked-out gene was considered essential if GR_ratio_ = 1 and nonessential if <1. Minimization of Metabolic Adjustment (MOMA [33]) algorithm was chosen to perform such analyses.

### 2.11. COG Analyses

The WebMGA web server [34] was used to provide functional Cluster of Orthologous Genes (COG) annotations (*p*-value cutoff of 0.001) to each gene in the model. The COG annotation for each gene associated with variable reactions during the transition between two niches was extracted from the WebMGA server. Biases were determined after standardizing by the number of genes in each class of variable genes. Statistical significance was determined using Pearson’s Chi-squared tests. The complete list of COG annotations is available as Supplementary Data 6.

## 3. Results

### 3.1. Validation of a Genome-Scale Metabolic Model of P. parmentieri SCC3193

FBA was employed to test if the reconstructed model could accurately predict the ability of *P. parmentieri* to produce biomass on utilized carbon sources. In particular, after verifying their presence in the metabolic reconstruction, we tested 91 compounds previously used in PM experiment. Details on the predictions of metabolites usage (before and after refinements) are given in Supplementary Data 1. Agreement between PM and in silico results is marked as TP (true positive) and TN (true negative), while discordance is marked as FP (false positive) and FN (false negative). The majority of the initial FN was solved by just adding an exchange reaction to the draft model.

**Figure 1:**
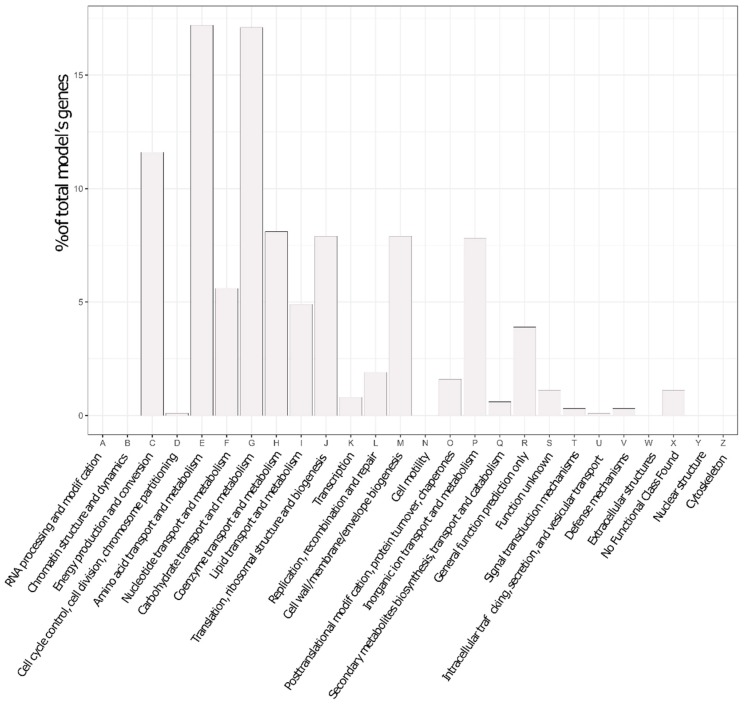
Genetic features captured by the model, according to Cluster of Orthologous Genes (COG) categories.

At the end of that refinement round, the final *P. parmentieri* model was termed iLP1245, by the nomenclature standard [35]. It includes 1245 genes (covering ~28% of the total number of coding sequences in the genome, 4449), 2182 reactions, and 2080 metabolites. Importantly, such coverage is generally accepted for metabolic models, since they only represent the fraction of metabolic genes. A description of the model is reported in Table 1 and the genetic features captured within it are presented as a plot of abundance per COG category in Figure 1.

**Figure 2:**
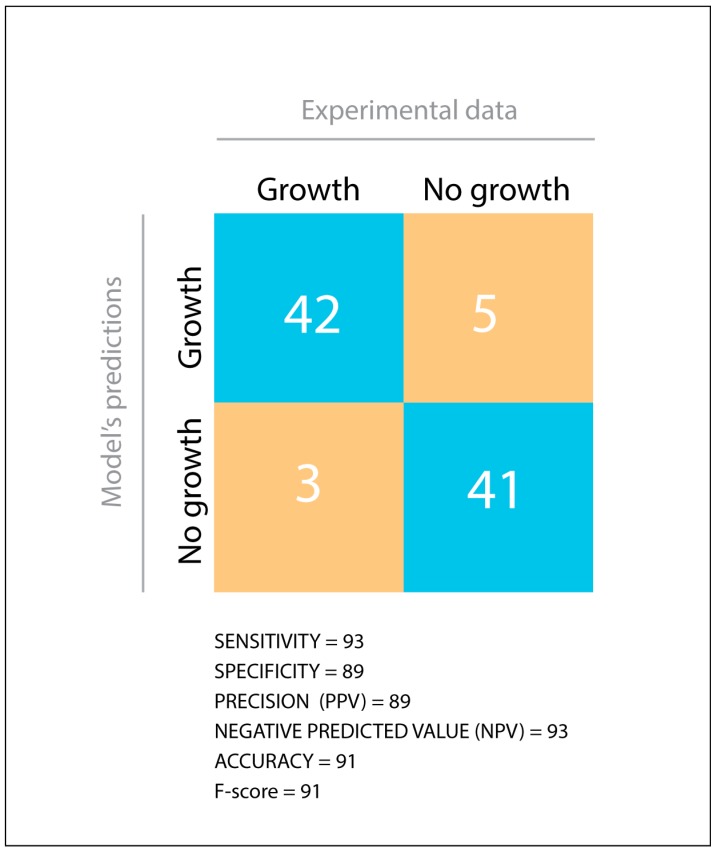
Comparison between Phenotype Microarray data and the model’s predictions. TP = True Positive, TN = True Negative, FP = False Positive, FN = False Negative. Statistical parameters were calculated as described in materials and methods.

During in silico assessment, iLP1245 displayed agreement with PM in 83 out of 91 tested carbon substrates. As summarized in Figure 2, sensitivity, specificity, precision, accuracy, negative predictive value, and *F*-score (calculated as described previously [36], see materials and methods) reached very high scores, suggesting high reliability of the model.

Systems Biology, providing a unique, unambiguous, perennial, standard-compliant, and directly resolvable Markup Language (SBML) file of the model was validated by the online SBML validator tool (http://sbml.org/Facilities/Validator/), and is available as Supplementary Data 2. All the metabolites embedded in the model were annotated by using identifiers.org and the MIRIAM [37] registry to facilitate model reuse and search strategies, by identifiers.

### 3.2. Phenotypic Characterization of P. parmentieri SCC3193 and Metabolic Model In-Depth Validation

Phenotypical profiling with the use of Biolog Plates PM1, PM2A, PM3, and PM4 revealed that *P. parmentieri* SCC3193 could utilize all common sugar components of plant cell walls at high levels, e.g., sucrose, tartaric acid, d-cellobiose, stachyose, and, most importantly, pectin (Poly(1,4-alpha-d-galacturonide)) (Supplementary Data 1, Supplementary Data 5). The bacterium was very effectively exploiting d-Glucosamine and its derivatives (*N*-Acetyl-d-Glucosamine, *N*-Acetyl-d-Galactosamine) together with xanthine (Supplementary Data 5). Khayi et al. 2016 [9] have proven that *P. parmentieri* strains can produce acid from raffinose and melibiose but not from malonic acid, and we also confirmed its ability to utilize raffinose and melibiose but not malonic acid by *P. parmentieri* in Biolog experiments (Supplementary Data 4). Besides, data about lactose, galactose, and maltose utilization were confirmed both in Biolog experiments and in silico simulations (Supplementary Data 1, Supplementary Data 4). Those results also agree with experimental data provided by Khayi et al. 2016 [9]. Moreover, metabolic abilities shown during PM experiments were compared to those obtained for other Enterobacteriaceae bacteria, including *E. coli* K12 MG1655, *Pectobacterium carotovorum* PC1 reported previously [26]. A comparison between these results is embedded in the Supplementary Data 1 file, and it highlights the metabolic similarity between the two *Pectobacterium* strains (80.53% of agreement) and *E. coli* (71.06% of agreement). Also, these findings support the biomass of *P. parmentieri* being approximated to that of *E. coli* (see materials and methods). We have also established of growth curves of *P. parmentieri* SCC3193 with randomly chosen carbon sources also analyzed in PM Microarray experiment, namely M9 medium supplemented with α-d-glucose, d-fructose, and d-xylose. In general, the obtained results agree with the qualitative model’s predictions (Supplementary Figure S1 and Supplementary Data 1).

### 3.3. Differential Metabolic Adaptation to Soil and Rhizosphere Environment

By analyzing flux changes in response to simulated environmental conditions (e.g., M9, soil, and the rhizosphere), it is possible to observe whether any significant metabolic rewiring occurs. Loopless FBA and loopless FVA (see Section 2) were used to estimate niche-specific metabolic adaptations in soil and the rhizosphere. The results obtained through FBA were cross-checked with FVA (see Supplementary Data 3 for results). Possible fluxes variations detected in the rhizosphere in respect to soil were interpreted as metabolic tuning occurring in the bacterium when it moves from one niche to the other. We focused only on the results for which variation is higher than 50% in rhizosphere compared to soil, as it was presented in a previous report [25].

**Figure 3:**
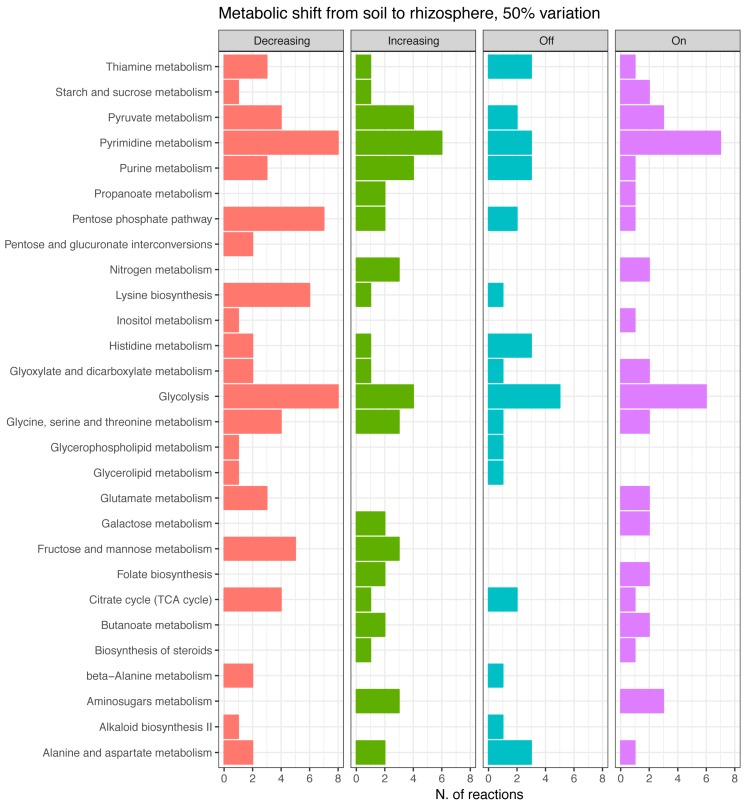
Reactions per metabolic pathways involved in the bacterial adaptation from soil to the rhizosphere. Flux changes higher than 50 % are classified into four categories: decreasing, increasing, turning off, turning on.

The total amount of reactions taken into account was 208, corresponding to ~10% of those embedded in the reconstruction. These were further classified in reactions with increased/decreased flux, and reactions turned on/off because of the environmental change (Figure 3). Notably, in this paper, we only present variations higher than 50%; however, the number of reactions changing flux was estimated at several cutoffs, i.e., 10%, 20%, 30%, 40%, and 50% of variation from the initial flux value (see Supplementary Figure S1 in Supplementary Information 2). Results indicate that the total number of reactions significantly changing the flux is slightly affected by the stringency of the cutoff, going from a maximum of 248 at 10% to a minimum of 208 at 50 % cutoff, thus vouching for robustness of the analysis.

Based on that criterion, it appears that several pathways remain active in both examined conditions, though recruiting a different set of reactions. The primary distinction seems to occur in sugar metabolic pathways (Figure 3), tracking a metabolic adjustment from hexose and pentose phosphate to amino sugars’ metabolism (amino sugars indeed are supposed to be more abundant in the rhizosphere [38]). Consequently, we can suggest that adaptation to the rhizosphere involves utilization of these carbon compounds [39] and that rhizosphere represents a rich environment for *P. parmentieri*, where these bacteria can thrive.

Also, some peculiar systems are turned on (Figure 3) in concomitance of such niche change, including nitrogen, butanoate, galactose, and propanoate metabolism, and biosynthetic pathways, including those of steroids and folate, indicating a specific adaptive response of *P. parmentieri* SCC3193 to the disparity of environmental (nutritional) setting. These compounds can either be precursors for volatiles compounds (VOCs) production or enter in VOC metabolic pathways; e.g., butanoate is an ester of butyric acid, which is among the most frequently secreted compounds [18,40]. VOCs could be strictly connected either with interbacterial communications or with virulence of plant pathogenic bacteria [18,40]. If so, this info could be used to develop pathogen biocontrol strategies.

#### Soil and Rhizosphere Adaptation, a Comparison with the Sinorhizobium meliloti Model

iLP1245 results in soil and rhizosphere were compared to those of the *Sinorhizobium meliloti* model [25], a plant symbiotic nitrogen-fixing bacterium, which, so far, is the only available GEM to be tested in such media. We observed that in *P. parmentieri* SCC3193, only 10% of reactions undergo a 50% flux change when comparing soil vs. rhizosphere; however, in *S. meliloti,* in the same conditions, more than the 20% of flux reactions was affected (including reactions which reversed flux direction). Moreover, in *S. meliloti*, ~13% of active reactions were specific to just one of the environments, while only 5.3% of active reactions were environment-specific in *P. parmentieri*.

We can hypothesize that the smaller and more compact genome of *P. parmentieri* compared to the *S. meliloti* genome (4449 vs. 6204 protein-coding genes, respectively, including a multipartite genome organization in the latter species) allows a reduced metabolic redundancy for the first compared to the second [41] and a more generalist vs. specialist metabolic network (i.e., most reactions are not changing while the environment fluctuates). To test this hypothesis, we performed MOMA simulations of gene deletions (see Supplementary Data 4). The analyses revealed that *P. parmentieri* SCC3193 possesses an essential gene core composed of 241 metabolic genes, allowing growth in both soil and rhizosphere. Here, a set of eight genes was found to be essential in soil but not in the rhizosphere. This is in vast contrast to *S. meliloti*, where 66 genes were found as essential for growth in the same simulated rhizosphere environment [25], and supports the previously proposed hypothesis of a robust metabolic network of *P. parmentieri* SCC3193, which may allow the strain to rapidly accommodate relevant changes in environmental nutrient sources. Additionally, we can assume that the dense core of essential genes important for *P. parmentieri* allows bacteria of this species to quickly and effectively adapt to changing environmental conditions. This might explain why bacteria from this species can persist on plant residuals without interacting with host plant (potato) for extended periods, and subsequently, also the cosmopolitan lifestyle of this bacteria in the environment [10].

### 3.4. In Silico Gene Deletions Provide Insight into the Fitness Relevance of Metabolic Modules

Simulated single gene deletion is a very powerful in silico method for estimating a gene’s knock-out fatality in as many settings as desired. Simulations on iLP1245 were made by using MOMA algorithm in three different media.

**Figure 4:**
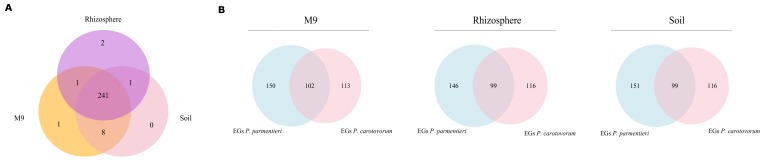
(**A**) Venn diagrams showing the amount of shared and unique essential genes in *P. parmentieri* SCC3193 for each of the examined conditions (M9, rhizosphere, and soil). (**B**) Venn diagrams showing the amount of shared and unique essential genes predicted by *P. carotovorum* PC1 model and *P. parmentieri* SCC3193 model for each of the examined conditions (M9, rhizosphere, and soil).

The number of essential genes found is 251 in M9 medium, 250 in soil, and 245 in the rhizosphere. The locus tag of such genes, alongside with their corresponding encoded protein, are reported in Supplementary Data 4. The Venn diagrams in Figure 4A show the overlap of EGs predicted among the three different conditions. Results indicate that a huge core of genes is likely to be mandatory in all the tested media, while only a small number stand out as essential in just one or two out of the three tested media. The method predicted the gene W5S_RS13875, encoding for DNA starvation/stationary phase protection protein (WP_014700482.1), as specifically required during growth in soil and rhizosphere, the gene W5S_RS15765, encoding for an ammonium transporter (WP_014700821.1), essential in M9 and rhizosphere, while eight genes appear to be specifically essential during growth in soil and M9 but not in rhizosphere. Seven of them belong to the thiamine and sulfur metabolism pathway: W5S_RS00965 (cystathionine gamma-synthase, WP_014698476.1), W5S_RS01140 (thiazole synthase ThiG, WP_012822036.1), W5S_RS01145 (sulfur carrier protein ThiS, WP_014698484.1), W5S_RS01150 (adenylyltransferase ThiF, WP_014698485.1), W5S_RS01155 (thiamine phosphate synthase ThiE, WP_014698486.1), W5S_RS01160 (phosphomethylpyrimidine synthase ThiC, WP_014698487.1), W5S_RS05940 (hydroxymethylpyrimidine/phosphomethylpyrimidine kinase ThiD, WP_014698998.1), while the last one, W5S_RS18250 (diaminopimelate decarboxylase, WP_043899153.1) is an enzyme involved in secondary metabolite production. Interestingly, the simulated rhizosphere growth medium contains thiamine which, conversely, is absent in soil and M9.

Three condition-specific genes have been predicted, one during growth in M9 medium, W5S_RS19605, encoding for class II fructose-bisphosphate aldolase (WP_014701539.1) and two during growth in the rhizosphere, W5S_RS19610 and W5S_RS06525, respectively encoding for a phosphoglycerate kinase (WP_005973111.1) and a long-chain fatty acid transporter (WP_014699104.1).

Moreover, we evaluated how the predicted EGs in iLP1245 compared to those predicted by the GEM reconstruction of *Pectobacterium carotovorum* PC1 [26], a closely related strain. Despite comparing EGs in different nutritional environments, we found a high level of overlap between the results given by the two models, as shown by Venn diagrams in Figure 4B. This supports the validity of EGs found through our simulation and, more in general, iLP1245 predictions consistency. As shown in Figure 4 and Supplementary Data 4, 99 genes are likely to be essential in *P. parmentieri* in all the simulated conditions, while seven are specifically essential in M9.

Importantly, we found out that nine genes, predicted as essentials by both models (listed in Table 2), were already marked as targets for new plants pathogens bactericides, since they share low sequence similarity both with their plant hosts and humans, ensuring safety requirement for agricultural field usage [26]. Moreover, these candidate targets are present in the therapeutic target database (TTD) [42], meaning that they are targets successfully used for human and animal pathogens, although they still have not been considered for crop disease treatments.

Since these nine genes are likely to be essential in both *P. parmentieri* SCC3193 and *P. carotovorum* subsp. *carotovorum* PC1, their essentiality should be tested in multiple *Pectobacterium*-like strains.

## 4. Conclusions

Plant–microbe interactions have been under intensive investigation in recent years, regardless of the nature of this communication: pathogenic or symbiotic. The ability to understand this interplay is connected with the complexity of the environment in which bacteria persist: soil, rhizosphere, or plant tissues. Metabolic modeling allows predicting and examining biochemical reactions involved in adaptation to the above-mentioned ecological niches as well as predicting the phenotypic outcomes of gene deletions. In this paper, for the first time, we report a high throughput experimental validation on the metabolic capability of *P. parmentieri* SCC3193 and a manually curated genome-scale metabolic model (GEM) of this plant pathogenic bacterium (iLP1245).

iLP1245 is highly reliable, obtaining 91% overlap in silico with large scale experimental data on carbon utilization phenotypes. This value perfectly fits with the currently accepted standard for GEMs [43,44]; for example, *Pectobacterium carotovorum* PC1 GEM showed an agreement of 80.4% with Phenotype Microarray (Biolog) experiments [26].

Moreover, we presented a broad set of EGs to be experimentally tested, nine of which are very likely to be used for bactericide design. This last result is significant since, at the moment, there is no available, efficient method for plant pathogenic bacteria eradication.

## Figures and Tables

**Table 1:** Properties of *P. parmentieri* SCC3193 genome and model.

*P. parmentieri* SCC3193 *Genome*	
Total genome size	4821
Total protein-coding genes	4449
Pseudo Genes	266
*iLP1245 characteristics*	
Total genes of *P. parmentieri* SCC3193	1245
Genes	1224
Pseudo Genes	21
Gap-filling genes	7
Total reactions	2182
Gene-associated reactions	2120
Exchange reactions	195
Transport reactions	324
Spontaneous reactions	55
Total metabolites	2080

**Table 2:** Candidate gene targets with no homology in humans and host and available in the therapeutic target database (TTD).

Locus Gene	Candidate Drug Targets	Function	EC No.
W5S_RS00975	WP_012822018.1	5,10-methylenetetrahydrofolate reductase	1.5.1.20
W5S_RS01035	WP_012822025.1	UDP-N-acetylenolpyruvoylglucosamine reductase	1.1.1.158
W5S_RS01045	WP_012822027.1	pantothenate kinase	2.7.1.33
W5S_RS09185	WP_014699609.1	GTP cyclohydrolase I	3.5.4.16
W5S_RS10395	WP_014699824.1	NAD synthetase	6.3.1.5
W5S_RS13505	WP_014700413.1	Thymidylate kinase	2.7.4.9
W5S_RS06665	WP_014699128.1	bifunctional folylpolyglutamate synthase/ dihydrofolate synthase	6.3.2.12/6.3.2.17
W5S_RS18995	WP_014701427.1	UDP-diphospho-muramoylpentapeptide beta-N-acetylglucosaminyltransferase	2.4.1.227
W5S_RS19920	WP_014701595.1	methionine synthase	2.1.2.13
W5S_RS20855	WP_005969274.1	aspartate-semialdehyde dehydrogenase	1.2.1.11
W5S_RS22025	WP_014701962.1	phosphopantetheine adenylyltransferase	2.7.7.3

## Supplementary Materials

The following are available online at https://www.mdpi.com/2076-2607/7/4/101/s1, **Supplementary Figure S1.** Growth curves and rates of *P. parmentieri* SCC3193. A: Growth curves of *P. parmentieri* SCC3193 in M9 media supplemented with different carbon sources over time *in vitro*. B: Growth rates of *P. parmentieri* SCC3193 obtained *in vitro* compared to in silico calculated growth rates basing on the metabolic model. **Supplementary Information 1.** The script is enabling to perform all the analysis in the manuscript. File type: python script. **Supplementary Information 2.** Supplementary Text, Supplementary Tables, and Supplementary Figures. File type: PDF document. **Supplementary Data 1.** Prediction on the usage of metabolites before and after the gap filling process. List of all reactions added/changed in the model during curation phases. Comparison of PM results between Escherichia coli K-12 MG1655, *Pectobacterium carotovorum* PC1, and *Pectobacterium parmentieri* SC3193. File type: Excel document. **Supplementary Data 2.** The SBML file of the model. File type: XML formatted file. **Supplementary Data 3.** Reactions Flux during growth in soil versus the rhizosphere according to loopless FBA and loopless FVA; File type: Excel document. **Supplementary Data 4.** All essential genes found with their encoded protein and function. Growth ratio calculated with FBA and MOMA is shown. EGs predicted by both *Pectobacterium carotovorum* PC1 model and *Pectobacterium parmentieri* SC3193 model. File type: Excel document. **Supplementary Data 5.** All Phenotype Microarray data generated in this study and dataset and plots generated through DuctApe program. File type: Excel document. **Supplementary Data 6.** The COG annotations for all genes included in the model, as generated by WebMGA. File type: Excel document.
