# Supplementary material for: Metabolic Modeling of Pectobacterium parmentieri SCC3193 Provides Insights into Metabolic Pathways of Plant Pathogenic Bacteria
Source: Microorganisms. 2019 Apr 5;7(4):101. doi: 10.3390/microorganisms7040101 (PMC6518042; doi:10.3390/microorganisms7040101)
Supplement: Supplementary file 1 [file microorganisms-07-00101-s001.zip › Supplementary figure 1.pdf]

A

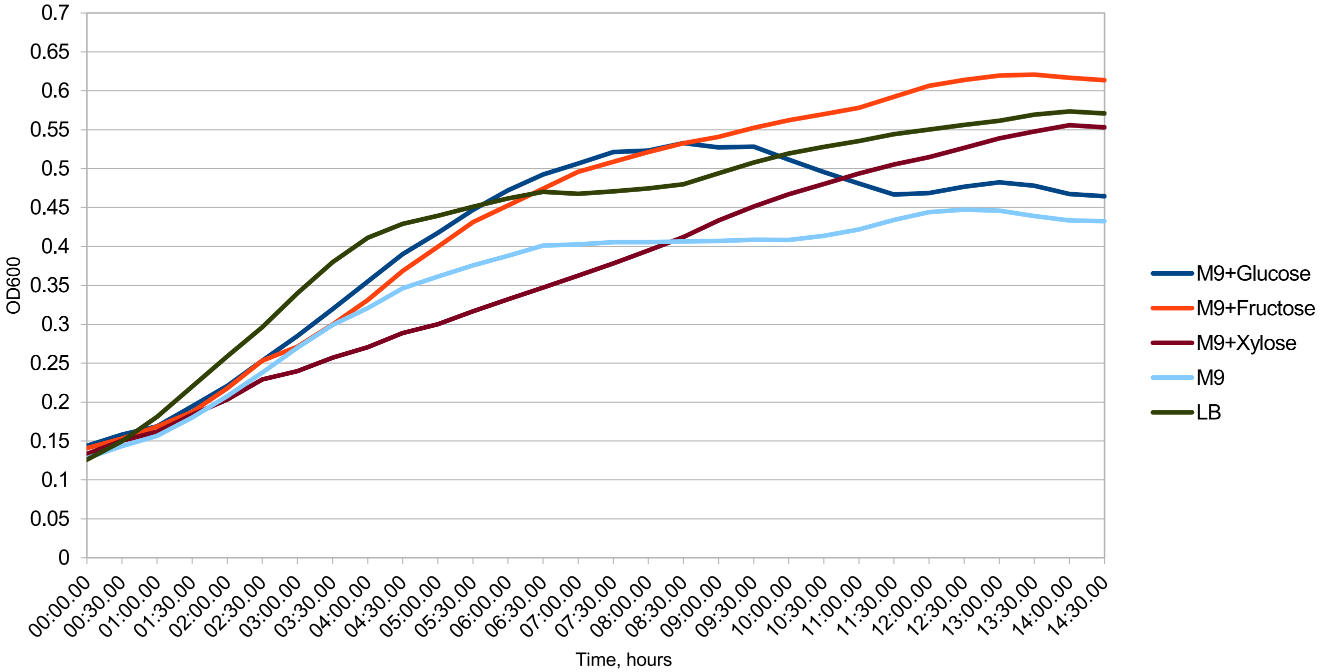

B

C- source	Experimental Growth Rate	Simulated uptake rate	BOF value yielded Insilico
Glucose	0.00576	-0,03	0.005
Fructose	0.00612	-0,004	0.006
Xylose	0.00548	-0,04	0.005
M9	0.00634	-0,08	0.006
